# 1-(2-Chloro­benzyl­idene)-2-(2,4-dinitro­phen­yl)hydrazine

**DOI:** 10.1107/S1600536808033357

**Published:** 2008-10-18

**Authors:** Zhi-Qiang Shi, Ning-Ning Ji, Xiao-Yan Li

**Affiliations:** a Department of Materials Science and Chemical Engineering, Taishan University, 271021 Taian, Shandong, People’s Republic of China; b Department of Chemistry, Taishan University, 271021 Taian, Shandong, People’s Republic of China; c No. 1 Middle School of Lanshan, 276808 Rizhao, Shandong, People’s Republic of China

## Abstract

In the title compound, C_13_H_9_ClN_4_O_4_, there are two crystallographically independent mol­ecules in the asymmetric unit, which have very similar conformations. The C=N—N angles in each independent mol­ecule are 115.0 (2) and 116.6 (2)°, which are significantly smaller than the ideal value of 120° expected for *sp* ^2^-hybridized N atoms. This is probably a consequence of repulsion between the nitro­gen lone pairs and the adjacent N—N bonds. Two bifurcated intra­molecular N—H⋯O hydrogen bonds help to establish the mol­ecular conformation and consolidate the crystal packing.

## Related literature

For general background, see: Garnovskii *et al.* (1993 ▶); Anderson *et al.* (1997 ▶); Musie *et al.* (2001 ▶); Paul *et al.* (2002 ▶); Shi *et al.* (2007 ▶); For related structures, see: Baughman *et al.* (2004 ▶); Zare *et al.* (2005 ▶); El-Seify & El-Dossoki (2006 ▶); Kim & Yoon (1998 ▶). For bond-length data, see: Allen *et al.* (1987 ▶).
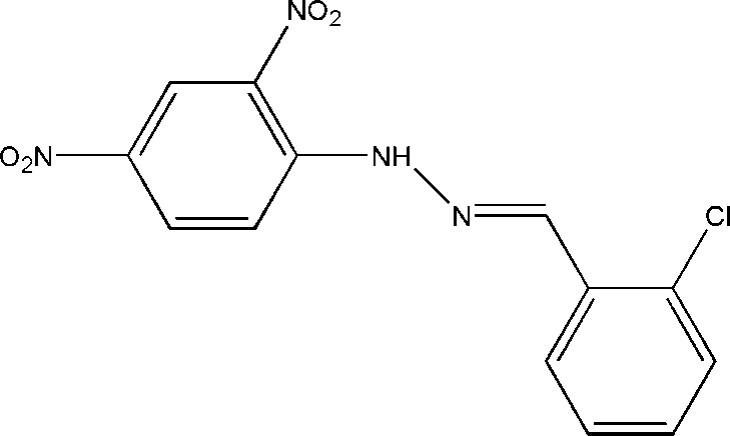


## Experimental

### 

#### Crystal data

C_13_H_9_ClN_4_O_4_
*M* *_r_* = 320.69
Triclinic, 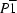

*a* = 7.2286 (7) Å
*b* = 7.6596 (8) Å
*c* = 25.145 (2) Å
α = 95.691 (2)°
β = 93.030 (2)°
γ = 99.728 (3)°
*V* = 1362.0 (2) Å^3^
*Z* = 4
Mo *K*α radiation
μ = 0.31 mm^−1^
*T* = 295 (2) K
0.15 × 0.12 × 0.08 mm

#### Data collection

Bruker APEXII CCD area-detector diffractometer
Absorption correction: multi-scan (*SADABS*; Bruker, 2005 ▶) *T*_min_ = 0.956, *T*_max_ = 0.976
7216 measured reflections
4776 independent reflections
3273 reflections with *I* > 2σ(*I*)
*R*_int_ = 0.021

#### Refinement

*R*[*F* ^2^ > 2σ(*F* ^2^)] = 0.047
*wR*(*F* ^2^) = 0.125
*S* = 1.07
4776 reflections
397 parameters
H-atom parameters constrained
Δρ_max_ = 0.42 e Å^−3^
Δρ_min_ = −0.36 e Å^−3^

### 

Data collection: *APEX2* (Bruker, 2005 ▶); cell refinement: *APEX2*; data reduction: *SAINT* (Bruker, 2005 ▶); program(s) used to solve structure: *SHELXTL* (Sheldrick, 2008 ▶); program(s) used to refine structure: *SHELXTL*; molecular graphics: *SHELXTL*; software used to prepare material for publication: *SHELXTL*.

## Supplementary Material

Crystal structure: contains datablocks global, I. DOI: 10.1107/S1600536808033357/fj2153sup1.cif

Structure factors: contains datablocks I. DOI: 10.1107/S1600536808033357/fj2153Isup2.hkl

Additional supplementary materials:  crystallographic information; 3D view; checkCIF report

## Figures and Tables

**Table 1:** Hydrogen-bond geometry (Å, °)

*D*—H⋯*A*	*D*—H	H⋯*A*	*D*⋯*A*	*D*—H⋯*A*
N6—H6⋯O6	0.86	2.02	2.631 (3)	127
N2—H2⋯O1	0.86	2.00	2.622 (3)	129

**Fig. 1.**
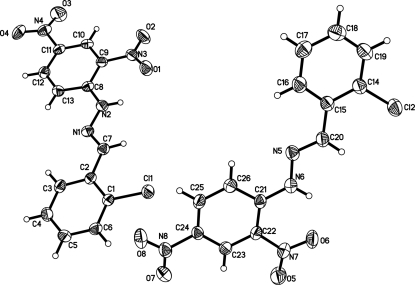
The molecular structure of (I), with displacement ellipsoids drawn at the 50% probability level.

**Fig. 2.**
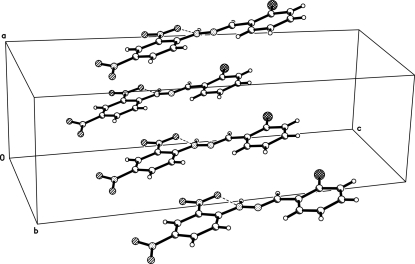
The molecules are stabilized by intramolecular N—H···O hydrogen bonds. The dashed lines indicate hydrogen bonds.

## supplementary crystallographic information

### Comment

In recent years, a number of Schiff-bases have been investigated in terms of their coordination chemistry (Garnovskii *et al.*, 1993; Musie *et al.*, 2001; Paul *et al.*, 2002; Shi *et al.*, 2007;) and biological systems (Anderson *et al.*, 1997). Especial the 2,4-dinitrophenylhydrazones exhibit good nonlinear optical (NLO) and crystalline properties (Baughman *et al.*, 2004). As a result of their significant molecular nonlinearities and remarkable ability to crystallize in non-centrosymmetric crystal systems (Zare *et al.*, 2005; El-Seify & El-Dossoki, 2006; Kim & Yoon, 1998), many X-ray structural studies of 2,4-dinitrophenylhydrazone have been reported. In order to search for new 2,4-dinitrophenylhydrazones, the title compound, (I), was synthesized and its crystal structure determined. In (I) (Fig. 1), the bond lengths and angles are in good agreement with the expected values (Allen *et al.*, 1987). In the crystal structure (Fig. 2), the molecules are stabilized by intramolecular N—H···O hydrogen bonds.

### Experimental

The title compound was synthesized by the reaction of (2,4-dinitro-phenyl)-hydrazine(1 mmol, 198.1 mg) with 2-Chloro-benzaldehyde (1 mmol, 140.6 mg) in ethanol (20 ml) under reflux conditions (343 K) for 3 h. The solvent was removed and the solid product recrystallized from tetrahydrofuran, and then dried *in vacuo* to give pure title compound in 89% yield. After five days yellow crystals suitable for X-ray diffraction study were obtained.

### Refinement

All H atoms were placed in idealized positions (C—H = 0.93–0.97 Å, N—H = 0.86 Å) and refined as riding atoms. For those bound to C, *U*_iso_(H) = 1.2 or 1.5*U*_eq_(C). while for those bound to N, *U*_iso_(H) = 1.2 *U*_eq_(N).

### Crystal data

**Table:**

C_13_H_9_ClN_4_O_4_	*Z* = 4
*M**_r_* = 320.69	*F*(000) = 656
Triclinic, *P*1	*D*_x_ = 1.564 Mg m^−^^3^
Hall symbol: -P 1	Mo *K*α radiation, λ = 0.71073 Å
*a* = 7.2286 (7) Å	Cell parameters from 1793 reflections
*b* = 7.6596 (8) Å	θ = 2.7–24.9°
*c* = 25.145 (2) Å	µ = 0.31 mm^−^^1^
α = 95.691 (2)°	*T* = 295 K
β = 93.030 (2)°	Block, yellow
γ = 99.728 (3)°	0.15 × 0.12 × 0.08 mm
*V* = 1362.0 (2) Å^3^	

### Data collection

**Table:**

Bruker APEXII CCD area-detector diffractometer	4776 independent reflections
Radiation source: fine-focus sealed tube	3273 reflections with *I* > 2σ(*I*)
graphite	*R*_int_ = 0.021
φ and ω scans	θ_max_ = 25.0°, θ_min_ = 2.5°
Absorption correction: multi-scan (SADABS; Bruker, 2005)	*h* = −8→8
*T*_min_ = 0.956, *T*_max_ = 0.976	*k* = −9→5
7216 measured reflections	*l* = −28→29

### Refinement

**Table:**

Refinement on *F*^2^	Primary atom site location: structure-invariant direct methods
Least-squares matrix: full	Secondary atom site location: difference Fourier map
*R*[*F*^2^ > 2σ(*F*^2^)] = 0.047	Hydrogen site location: inferred from neighbouring sites
*wR*(*F*^2^) = 0.125	H-atom parameters constrained
*S* = 1.07	*w* = 1/[σ^2^(*F*_o_^2^) + (0.0511*P*)^2^ + 0.2987*P*] where *P* = (*F*_o_^2^ + 2*F*_c_^2^)/3
4776 reflections	(Δ/σ)_max_ = 0.001
397 parameters	Δρ_max_ = 0.42 e Å^−^^3^
0 restraints	Δρ_min_ = −0.36 e Å^−^^3^

### Special details

**Table:**

Geometry. All e.s.d.'s (except the e.s.d. in the dihedral angle between two l.s. planes) are estimated using the full covariance matrix. The cell e.s.d.'s are taken into account individually in the estimation of e.s.d.'s in distances, angles and torsion angles; correlations between e.s.d.'s in cell parameters are only used when they are defined by crystal symmetry. An approximate (isotropic) treatment of cell e.s.d.'s is used for estimating e.s.d.'s involving l.s. planes.
Refinement. Refinement of *F*^2^ against ALL reflections. The weighted *R*-factor *wR* and goodness of fit *S* are based on *F*^2^, conventional *R*-factors *R* are based on *F*, with *F* set to zero for negative *F*^2^. The threshold expression of *F*^2^ > σ(*F*^2^) is used only for calculating *R*-factors(gt) *etc*. and is not relevant to the choice of reflections for refinement. *R*-factors based on *F*^2^ are statistically about twice as large as those based on *F*, and *R*- factors based on ALL data will be even larger.

### Fractional atomic coordinates and isotropic or equivalent isotropic displacement parameters (Å^2^)

**Table:**

	*x*	*y*	*z*	*U*_iso_*/*U*_eq_	
Cl1	0.32732 (10)	0.39535 (11)	0.21587 (3)	0.0647 (2)	
Cl2	0.43812 (11)	0.46461 (12)	0.65151 (3)	0.0739 (3)	
O1	1.1413 (3)	0.8066 (3)	0.24502 (8)	0.0769 (6)	
O2	1.4364 (3)	0.8957 (4)	0.23973 (8)	0.0901 (8)	
O3	1.6810 (3)	1.1413 (4)	0.08840 (11)	0.1047 (9)	
O4	1.4958 (3)	1.1774 (3)	0.02231 (9)	0.0754 (6)	
O5	−0.0518 (4)	−0.1695 (3)	0.37193 (10)	0.0957 (8)	
O6	0.0550 (3)	−0.0357 (3)	0.44708 (4)	0.0817 (7)	
O7	−0.1612 (4)	0.1254 (4)	0.22106 (9)	0.0899 (8)	
O8	−0.1463 (3)	0.4102 (3)	0.23032 (8)	0.0804 (7)	
N1	0.7500 (3)	0.6855 (3)	0.12714 (8)	0.0490 (5)	
N2	0.9138 (3)	0.7485 (3)	0.15793 (8)	0.0506 (6)	
H2	0.9218	0.7309	0.1912	0.061*	
N3	1.2749 (4)	0.8647 (3)	0.21971 (9)	0.0586 (6)	
N4	1.5265 (4)	1.1204 (3)	0.06490 (11)	0.0626 (7)	
N5	0.2250 (3)	0.4769 (3)	0.48803 (9)	0.0544 (6)	
N6	0.1464 (3)	0.3138 (3)	0.46212 (8)	0.0555 (6)	
H6	0.1359	0.2206	0.4790	0.067*	
N7	0.0038 (3)	−0.0334 (3)	0.40054 (10)	0.0584 (6)	
N8	−0.1249 (3)	0.2712 (4)	0.24773 (9)	0.0613 (6)	
C1	0.2909 (3)	0.4329 (3)	0.14910 (10)	0.0440 (6)	
C2	0.4356 (3)	0.5294 (3)	0.12368 (10)	0.0428 (6)	
C3	0.3991 (4)	0.5523 (4)	0.07027 (10)	0.0508 (7)	
H3	0.4928	0.6154	0.0522	0.061*	
C4	0.2273 (4)	0.4835 (4)	0.04363 (11)	0.0565 (7)	
H4	0.2058	0.5001	0.0078	0.068*	
C5	0.0867 (4)	0.3899 (4)	0.06992 (11)	0.0558 (7)	
H5	−0.0295	0.3438	0.0518	0.067*	
C6	0.1179 (4)	0.3646 (3)	0.12285 (11)	0.0506 (7)	
H6A	0.0232	0.3020	0.1407	0.061*	
C7	0.6178 (3)	0.6038 (3)	0.15160 (10)	0.0470 (6)	
H7	0.6378	0.5915	0.1878	0.056*	
C8	1.0634 (3)	0.8386 (3)	0.13611 (10)	0.0436 (6)	
C9	1.2406 (3)	0.8962 (3)	0.16462 (10)	0.0450 (6)	
C10	1.3931 (3)	0.9855 (3)	0.14090 (10)	0.0466 (6)	
H10	1.5096	1.0204	0.1599	0.056*	
C11	1.3680 (4)	1.0207 (3)	0.08927 (10)	0.0479 (6)	
C12	1.1976 (4)	0.9667 (4)	0.05948 (11)	0.0522 (7)	
H12	1.1849	0.9913	0.0241	0.063*	
C13	1.0483 (4)	0.8770 (3)	0.08255 (10)	0.0501 (7)	
H13	0.9341	0.8404	0.0625	0.060*	
C14	0.4405 (4)	0.6574 (4)	0.62064 (11)	0.0555 (7)	
C15	0.3642 (4)	0.6497 (4)	0.56796 (10)	0.0517 (7)	
C16	0.3711 (4)	0.8103 (4)	0.54624 (12)	0.0626 (8)	
H16	0.3214	0.8099	0.5113	0.075*	
C17	0.4496 (4)	0.9698 (5)	0.57508 (13)	0.0700 (9)	
H17	0.4537	1.0755	0.5595	0.084*	
C18	0.5230 (4)	0.9733 (5)	0.62741 (13)	0.0713 (9)	
H18	0.5753	1.0813	0.6471	0.086*	
C19	0.5181 (4)	0.8170 (5)	0.65008 (12)	0.0664 (8)	
H19	0.5669	0.8187	0.6851	0.080*	
C20	0.2786 (4)	0.4815 (4)	0.53736 (10)	0.0548 (7)	
H20	0.2629	0.3771	0.5539	0.066*	
C21	0.0852 (3)	0.2998 (4)	0.40979 (10)	0.0451 (6)	
C22	0.0134 (3)	0.1353 (3)	0.37897 (10)	0.0461 (6)	
C23	−0.0515 (3)	0.1268 (4)	0.32600 (10)	0.0482 (6)	
H23	−0.0975	0.0172	0.3064	0.058*	
C24	−0.0474 (3)	0.2805 (4)	0.30277 (9)	0.0462 (6)	
C25	0.0247 (4)	0.4448 (4)	0.33107 (10)	0.0512 (7)	
H25	0.0282	0.5484	0.3145	0.061*	
C26	0.0902 (4)	0.4542 (4)	0.38328 (10)	0.0513 (7)	
H26	0.1394	0.5649	0.4018	0.062*	

### Atomic displacement parameters (Å^2^)

**Table:**

	*U*^11^	*U*^22^	*U*^33^	*U*^12^	*U*^13^	*U*^23^
Cl1	0.0580 (4)	0.0837 (5)	0.0489 (4)	−0.0052 (4)	0.0051 (3)	0.0196 (4)
Cl2	0.0731 (5)	0.0892 (6)	0.0596 (5)	0.0129 (4)	−0.0076 (4)	0.0178 (4)
O1	0.0683 (14)	0.1072 (18)	0.0515 (12)	−0.0034 (13)	0.0101 (10)	0.0190 (12)
O2	0.0658 (15)	0.131 (2)	0.0598 (13)	−0.0178 (14)	−0.0154 (11)	0.0137 (13)
O3	0.0540 (14)	0.142 (2)	0.1053 (19)	−0.0302 (15)	0.0087 (14)	0.0299 (17)
O4	0.0938 (17)	0.0597 (13)	0.0773 (15)	0.0076 (12)	0.0342 (12)	0.0249 (12)
O5	0.158 (3)	0.0482 (13)	0.0751 (16)	0.0065 (15)	−0.0070 (15)	0.0038 (12)
O6	0.123 (2)	0.0665 (14)	0.0573 (13)	0.0157 (13)	−0.0011 (13)	0.0240 (11)
O7	0.110 (2)	0.0898 (18)	0.0566 (13)	−0.0106 (15)	−0.0197 (13)	0.0052 (13)
O8	0.0857 (16)	0.1023 (18)	0.0622 (13)	0.0302 (14)	−0.0007 (11)	0.0327 (13)
N1	0.0405 (12)	0.0503 (13)	0.0541 (13)	0.0000 (10)	0.0056 (10)	0.0075 (11)
N2	0.0407 (12)	0.0596 (14)	0.0483 (12)	−0.0026 (11)	0.0055 (10)	0.0087 (11)
N3	0.0588 (15)	0.0641 (16)	0.0468 (13)	−0.0031 (13)	0.0014 (12)	0.0015 (11)
N4	0.0632 (17)	0.0517 (15)	0.0701 (17)	−0.0032 (13)	0.0235 (14)	0.0057 (13)
N5	0.0539 (14)	0.0647 (16)	0.0447 (13)	0.0106 (12)	0.0054 (11)	0.0056 (12)
N6	0.0634 (15)	0.0572 (15)	0.0458 (13)	0.0082 (12)	0.0013 (11)	0.0114 (11)
N7	0.0695 (16)	0.0589 (16)	0.0513 (15)	0.0147 (13)	0.0099 (12)	0.0185 (13)
N8	0.0507 (14)	0.086 (2)	0.0468 (14)	0.0045 (14)	0.0041 (11)	0.0180 (15)
C1	0.0436 (14)	0.0430 (14)	0.0459 (14)	0.0060 (12)	0.0066 (11)	0.0081 (12)
C2	0.0395 (14)	0.0403 (14)	0.0508 (15)	0.0089 (11)	0.0069 (11)	0.0109 (12)
C3	0.0467 (15)	0.0534 (16)	0.0559 (16)	0.0097 (13)	0.0110 (13)	0.0187 (13)
C4	0.0537 (17)	0.0669 (19)	0.0502 (16)	0.0106 (15)	−0.0001 (13)	0.0155 (14)
C5	0.0441 (15)	0.0583 (18)	0.0625 (18)	0.0037 (13)	−0.0058 (13)	0.0088 (14)
C6	0.0418 (15)	0.0514 (16)	0.0577 (17)	0.0015 (13)	0.0051 (12)	0.0120 (13)
C7	0.0423 (14)	0.0480 (16)	0.0512 (15)	0.0060 (12)	0.0058 (12)	0.0093 (13)
C8	0.0425 (14)	0.0393 (14)	0.0478 (15)	0.0030 (12)	0.0095 (11)	0.0026 (11)
C9	0.0478 (15)	0.0415 (14)	0.0425 (14)	0.0007 (12)	0.0047 (12)	0.0006 (11)
C10	0.0399 (14)	0.0411 (15)	0.0544 (16)	−0.0018 (12)	0.0038 (12)	−0.0015 (12)
C11	0.0480 (16)	0.0392 (14)	0.0545 (16)	−0.0011 (12)	0.0132 (13)	0.0049 (12)
C12	0.0544 (17)	0.0531 (17)	0.0491 (15)	0.0055 (14)	0.0079 (13)	0.0095 (13)
C13	0.0446 (15)	0.0510 (16)	0.0519 (16)	0.0013 (13)	0.0005 (12)	0.0058 (13)
C14	0.0407 (15)	0.075 (2)	0.0496 (16)	0.0070 (14)	0.0036 (12)	0.0078 (15)
C15	0.0413 (15)	0.0687 (19)	0.0446 (15)	0.0058 (14)	0.0084 (12)	0.0073 (14)
C16	0.0589 (18)	0.077 (2)	0.0512 (17)	0.0058 (16)	0.0117 (14)	0.0091 (16)
C17	0.067 (2)	0.069 (2)	0.075 (2)	0.0072 (17)	0.0198 (17)	0.0153 (18)
C18	0.0606 (19)	0.078 (2)	0.070 (2)	0.0016 (17)	0.0130 (16)	−0.0064 (18)
C19	0.0544 (18)	0.089 (2)	0.0519 (17)	0.0091 (17)	0.0009 (14)	−0.0013 (18)
C20	0.0517 (16)	0.069 (2)	0.0442 (16)	0.0088 (15)	0.0039 (13)	0.0126 (14)
C21	0.0395 (14)	0.0584 (17)	0.0399 (14)	0.0102 (13)	0.0087 (11)	0.0115 (13)
C22	0.0455 (15)	0.0498 (16)	0.0459 (15)	0.0095 (13)	0.0096 (12)	0.0142 (13)
C23	0.0443 (15)	0.0559 (17)	0.0440 (15)	0.0046 (13)	0.0080 (11)	0.0074 (13)
C24	0.0393 (14)	0.0624 (18)	0.0375 (14)	0.0067 (13)	0.0046 (11)	0.0108 (13)
C25	0.0495 (16)	0.0574 (18)	0.0499 (16)	0.0083 (14)	0.0100 (12)	0.0202 (14)
C26	0.0513 (16)	0.0526 (16)	0.0499 (16)	0.0066 (13)	0.0063 (12)	0.0082 (13)

### Geometric parameters (Å, °)

**Table:**

Cl1—C1	1.745 (2)	C5—H5	0.9300
Cl2—C14	1.733 (3)	C6—H6A	0.9300
O1—N3	1.229 (3)	C7—H7	0.9300
O2—N3	1.222 (3)	C8—C13	1.409 (3)
O3—N4	1.214 (3)	C8—C9	1.413 (3)
O4—N4	1.220 (3)	C9—C10	1.391 (3)
O5—N7	1.202 (3)	C10—C11	1.361 (3)
O6—N7	1.211 (3)	C10—H10	0.9300
O7—N8	1.224 (3)	C11—C12	1.385 (4)
O8—N8	1.221 (3)	C12—C13	1.367 (3)
N1—C7	1.272 (3)	C12—H12	0.9300
N1—N2	1.368 (3)	C13—H13	0.9300
N2—C8	1.354 (3)	C14—C19	1.379 (4)
N2—H2	0.8600	C14—C15	1.399 (4)
N3—O1	1.229 (3)	C15—C16	1.389 (4)
N3—C9	1.445 (3)	C15—C20	1.456 (4)
N4—C11	1.464 (3)	C16—C17	1.374 (4)
N5—C20	1.275 (3)	C16—H16	0.9300
N5—N6	1.367 (3)	C17—C18	1.389 (4)
N6—C21	1.354 (3)	C17—H17	0.9300
N6—H6	0.8600	C18—C19	1.372 (4)
N7—O6	1.211 (3)	C18—H18	0.9300
N7—C22	1.443 (3)	C19—H19	0.9300
N8—C24	1.456 (3)	C20—H20	0.9300
C1—C6	1.377 (3)	C21—C26	1.410 (4)
C1—C2	1.396 (3)	C21—C22	1.414 (4)
C2—C3	1.389 (3)	C22—C23	1.380 (3)
C2—C7	1.457 (3)	C23—C24	1.362 (4)
C3—C4	1.375 (4)	C23—H23	0.9300
C3—H3	0.9300	C24—C25	1.386 (4)
C4—C5	1.379 (4)	C25—C26	1.363 (4)
C4—H4	0.9300	C25—H25	0.9300
C5—C6	1.376 (4)	C26—H26	0.9300
			
C7—N1—N2	115.0 (2)	C11—C10—C9	118.5 (2)
C8—N2—N1	120.0 (2)	C11—C10—H10	120.7
C8—N2—H2	120.0	C9—C10—H10	120.7
N1—N2—H2	120.0	C10—C11—C12	122.1 (2)
O2—N3—O1	121.8 (2)	C10—C11—N4	118.6 (2)
O2—N3—O1	121.8 (2)	C12—C11—N4	119.3 (2)
O2—N3—C9	118.9 (2)	C13—C12—C11	119.4 (2)
O1—N3—C9	119.2 (2)	C13—C12—H12	120.3
O1—N3—C9	119.2 (2)	C11—C12—H12	120.3
O3—N4—O4	123.9 (3)	C12—C13—C8	121.5 (2)
O3—N4—C11	117.9 (3)	C12—C13—H13	119.3
O4—N4—C11	118.2 (3)	C8—C13—H13	119.3
C20—N5—N6	116.6 (2)	C19—C14—C15	121.8 (3)
C21—N6—N5	119.6 (2)	C19—C14—Cl2	117.5 (2)
C21—N6—H6	120.2	C15—C14—Cl2	120.7 (2)
N5—N6—H6	120.2	C16—C15—C14	117.1 (3)
O5—N7—O6	120.9 (2)	C16—C15—C20	121.2 (3)
O5—N7—O6	120.9 (2)	C14—C15—C20	121.7 (3)
O5—N7—C22	119.6 (2)	C17—C16—C15	121.6 (3)
O6—N7—C22	119.4 (3)	C17—C16—H16	119.2
O6—N7—C22	119.4 (3)	C15—C16—H16	119.2
O8—N8—O7	123.5 (3)	C16—C17—C18	120.0 (3)
O8—N8—C24	118.0 (3)	C16—C17—H17	120.0
O7—N8—C24	118.5 (3)	C18—C17—H17	120.0
C6—C1—C2	121.8 (2)	C19—C18—C17	119.8 (3)
C6—C1—Cl1	118.19 (19)	C19—C18—H18	120.1
C2—C1—Cl1	119.98 (19)	C17—C18—H18	120.1
C3—C2—C1	117.3 (2)	C18—C19—C14	119.7 (3)
C3—C2—C7	120.8 (2)	C18—C19—H19	120.2
C1—C2—C7	121.9 (2)	C14—C19—H19	120.2
C4—C3—C2	121.3 (2)	N5—C20—C15	120.2 (3)
C4—C3—H3	119.4	N5—C20—H20	119.9
C2—C3—H3	119.4	C15—C20—H20	119.9
C3—C4—C5	120.1 (3)	N6—C21—C26	120.1 (3)
C3—C4—H4	120.0	N6—C21—C22	123.4 (2)
C5—C4—H4	120.0	C26—C21—C22	116.5 (2)
C6—C5—C4	120.2 (3)	C23—C22—C21	121.7 (2)
C6—C5—H5	119.9	C23—C22—N7	115.8 (2)
C4—C5—H5	119.9	C21—C22—N7	122.5 (2)
C1—C6—C5	119.3 (2)	C24—C23—C22	119.3 (3)
C1—C6—H6A	120.3	C24—C23—H23	120.4
C5—C6—H6A	120.3	C22—C23—H23	120.4
N1—C7—C2	120.9 (2)	C23—C24—C25	121.1 (2)
N1—C7—H7	119.6	C23—C24—N8	119.0 (3)
C2—C7—H7	119.6	C25—C24—N8	119.8 (2)
N2—C8—C13	120.4 (2)	C26—C25—C24	119.9 (2)
N2—C8—C9	122.8 (2)	C26—C25—H25	120.0
C13—C8—C9	116.8 (2)	C24—C25—H25	120.0
C10—C9—C8	121.7 (2)	C25—C26—C21	121.4 (3)
C10—C9—N3	116.1 (2)	C25—C26—H26	119.3
C8—C9—N3	122.3 (2)	C21—C26—H26	119.3

### Hydrogen-bond geometry (Å, °)

**Table:**

*D*—H···*A*	*D*—H	H···*A*	*D*···*A*	*D*—H···*A*
N6—H6···O6	0.86	2.02	2.631 (3)	127
N2—H2···O1	0.86	2.00	2.622 (3)	129
